# Advances in the behavioural testing and network imaging of rodent recognition memory

**DOI:** 10.1016/j.bbr.2014.07.049

**Published:** 2015-05-15

**Authors:** Lisa Kinnavane, Mathieu M. Albasser, John P. Aggleton

**Affiliations:** School of Psychology, Cardiff University, Tower Building, 70 Park Place, Cardiff, Wales CF10 3AT, United Kingdom

**Keywords:** Behavioural testing, Entorhinal cortex, Hippocampus, Neural network, Perirhinal cortex, Recognition memory

## Abstract

Research into object recognition memory has been galvanised by the introduction of spontaneous preference tests for rodents. The standard task, however, contains a number of inherent shortcomings that reduce its power. Particular issues include the problem that individual trials are time consuming, so limiting the total number of trials in any condition. In addition, the spontaneous nature of the behaviour and the variability between test objects add unwanted noise. To combat these issues, the ‘bow-tie maze’ was introduced. Although still based on the spontaneous preference of novel over familiar stimuli, the ability to give multiple trials within a session without handling the rodents, as well as using the same objects as both novel and familiar samples on different trials, overcomes key limitations in the standard task. Giving multiple trials within a single session also creates new opportunities for functional imaging of object recognition memory. A series of studies are described that examine the expression of the immediate-early gene, c-*fos*. Object recognition memory is associated with increases in perirhinal cortex and area Te2 c-*fos* activity. When rats explore novel objects the pathway from the perirhinal cortex to lateral entorhinal cortex, and then to the dentate gyrus and CA3, is engaged. In contrast, when familiar objects are explored the pathway from the perirhinal cortex to lateral entorhinal cortex, and then to CA1, takes precedence. The switch to the perforant pathway (novel stimuli) from the temporoammonic pathway (familiar stimuli) may assist the enhanced associative learning promoted by novel stimuli.

## Highlights

• History of behavioural testing for recognition memory in primates.
• Behavioural testing for recognition memory in rodents using spontaneous recognition.
• The functional imaging of rodent recognition memory using immediate-early genes.
• Network analyses based on structural equation modelling of immediate-early gene data.

## 1 Introduction

Recognition memory is the ability to distinguish novel from familiar stimuli. This ubiquitous form of memory is shared across animal species, making it an important target for neuroscientific investigation. However, in order to understand the neural basis of recognition memory in animals it is necessary to develop appropriate behavioural tests. This review first considers the historical development of behavioural tests of object recognition as this information helps to clarify the rationale behind current tests and explains the present, overwhelming reliance on just one particular class of test, namely spontaneous preference tests; see also [1–4]. In such tests, recognition memory is inferred by the greater lengths of time spent exploring novel rather than familiar stimuli. The review focusses on how object recognition memory has been tested in rodents, highlighting existing challenges and describing shortcomings inherent in the most popular tests of recognition memory. More recent procedures that are designed to overcome these shortcomings are then described. One consequence of these recent developments in behavioural testing is the enhanced ability to image networks of neural interactions associated with recognition memory. These imaging experiments are discussed in Section 3.

## 2 A brief history of behavioural testing for recognition memory

### 2.1 Delayed nonmatching-to-sample (DNMS)

**Fig. 1:**
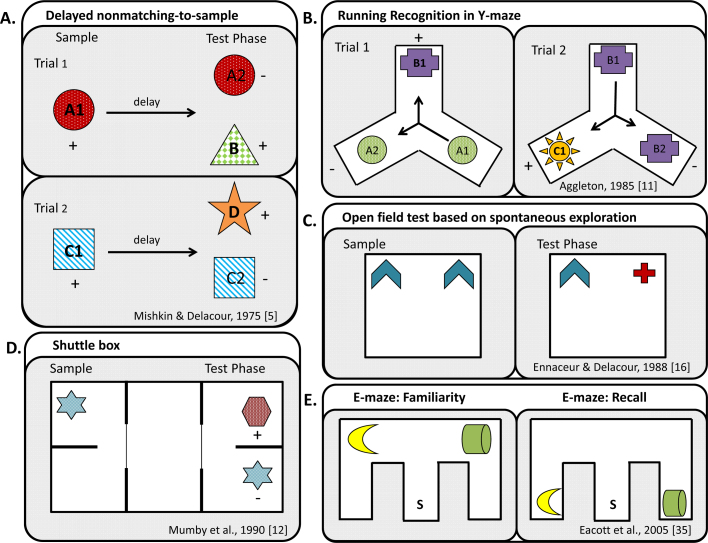
Schematic illustration of various tests of object recognition memory. (A) Delayed nonmatching-to-sample task designed for monkeys [5]. (B) Running Recognition (nonmatching-to-sample) in a Y-maze [11]; arrows show direction of rats’ movements. (C) Open field test of spontaneous object recognition memory [16]; none of the objects are associated with a food reward. (D) Shuttle box nonmatching-to-sample test [12]; two sliding doors separate the central holding area from the sample and test regions at the ends of the maze. (E) E-maze [35]; S denotes the start arm. Configuration of sample and test phases in the E-maze for both familiarity and recall are as shown. Upon completion of the sample phase the rat is placed in a holding cage with a copy of one of the objects from the sample phase for habituation. The animal is then returned to the maze: When the objects are placed on the backbone of the E-maze the rat can see them from the start arm and so can choose the non-habituated object based on familiarity processing whereas when the objects are placed in the outer arms they cannot be seen from the start arm and so the rat must use recall processes to remember where the non-habituated object is located. + symbol, objects associated with food reward. Bold letters represent novel objects.

It could be argued that one of the most important challenges in recognition memory research has been the problem of creating a behavioural test that closely mimics how recognition memory is tested in humans. The first such test was delayed nonmatching-to-sample (DNMS), which was introduced by Mishkin and Delacour [5]; see also [6]. The DNMS task is based on the discovery that, by using a rewarded forced-choice procedure, monkeys can rapidly be trained to select a novel object in preference to a familiar object, i.e., demonstrate recognition memory. Critically, the DNMS task only requires a single exposure phase in which to familiarise the initial sample stimulus, e.g., object A. After allowing the monkey to inspect object A, the monkey is given a choice, after a delay, between the now familiar object A and novel object B. Selection of novel object B is rewarded. In this way, the monkey is reinforced for applying a nonmatching rule to a familiar sample object after a retention delay, i.e., delayed nonmatching-to-sample. The next trial involves a completely new pair of objects, e.g., sample object C and novel object D. Thus, a series of trials can be represented as A+ then A− vs B+ (trial 1); C+ then C− vs D+ (trial 2); E+ then E− vs F+ (trial 3), and so on, where the object with a plus sign covers a food reward and the object with a negative sign is unrewarded (Fig. 1A).

The DNMS task not only has clear parallels with forced-choice recognition tests given to humans but also permits multiple recognition problems within a single session. As studies with monkeys inevitably rely on very small group sizes, the ability to give many trials per session is an essential feature if the task is going to differentiate between neural manipulations. The DNMS protocol also proved to be highly versatile as it can easily be given with varying retention intervals and altered levels of interference between sample and recognition test [7]. The task can also be given in the dark in order to test tactile recognition memory [8]. By taking advantage of the monkey's spontaneous preference for novelty over familiarity, the DNMS task is not only quick to train but reduces the likelihood that deficits arise because the nonmatching rule itself has been lost. Furthermore, it proved relatively straightforward to test humans on both delayed matching-to-sample and delayed nonmatching-to-sample tasks that were deliberately modelled on DNMS tests given to monkeys. Such experiments showed, for example, that anterograde amnesia is often sensitive to this form of recognition test [9,10].

Not surprisingly, the next step was to determine if rodents could also learn a delayed nonmatching-to-sample task that involved a single sample exposure. In the first such experiment [11], rats ran in a Y-shaped maze where they selected between objects using a ‘running recognition’ protocol (Fig. 1B). Consequently, the novel stimulus for one trial became the familiar stimulus for the next trial. In practice, the reinforced rule was to choose the arm with novel contents and avoid the arms with familiar contents (Fig. 1B). As the nonmatching procedure was continuous there was no discrete sample phase, apart from at the very start of the session (trial 0). The task design can, therefore, be represented as A+ (trial 0), A− vs B+ (trial 1), B− vs C+ (trial 2), D+ vs C− (trial 3), E+ vs D− (trial 4), and so on. The continuous testing procedure makes it possible to give multiple trials per session without having to handle the rats.

A later nonmatching task for rats [12] included a separate sample phase at the start of each trial, so more closely following the DNMS procedures given to monkeys. The apparatus consisted of a shuttle box with a central holding area [12] (Fig. 1D). One end of the apparatus was used for the sample phase while at the other end the rat was rewarded with food for selecting a novel object rather than the familiar sample object. To start each trial, the rat ran from the central holding area to the sample end to explore novel object A. This familiarisation phase was followed by a choice test at the opposite end of the shuttle box between the now familiar, object A and a novel alternative, object B. New sets of objects were used for each trial. Again, the rats were not handled during the test session and multiple trials could be given within a session [12].

Anyone involved in the behavioural testing of rodent recognition memory will know that these rodent DNMS tasks have been replaced by other, simpler methods. The problem is that rodent DNMS tasks involved considerable training. Even then, some rats struggled to reach levels of performance that would be informative when trying to manipulate recognition memory. A related issue was that because task acquisition was demanding, it remained possible that neural interventions might affect performance by altering the ability to learn and apply the rule, rather than by affecting the ability to distinguish novel from familiar stimuli. These tasks also depended on the use of food rewards, meaning changes in motivation might alter performance. A much simpler test was required.

### 2.2 Spontaneous object recognition

The assessment of recognition memory in rodents was transformed by the introduction of spontaneous preference tests based on measurements of exploration. It had been shown in T-maze studies that rats prefer arms with a novel appearance [13,14]. It had also been found that hamsters will spend more time exploring an object that has moved to a novel position [15]. Utilising this preference for novelty, Ennaceur and Delacour [16] showed that if rats are given sufficient time to explore object A in an open rectangular arena, they will typically spend more time exploring novel object B in preference to a duplicate of object A, when put back in that same arena (Fig. 1C). This simple, but powerful, protocol has led to countless experiments into the neural basis of recognition memory. The basic methodology has also proved to be remarkably versatile. Spontaneous preference tasks have been designed to measure memory for object location [15,17], object-in-place information [18], object-in-context conjunctions [18,19], object reconfigurations [20] and object recency [21], but see [2], along with various combinations of these forms of memory [22–24].

The attractions of the spontaneous object recognition task are obvious. The task rule is spontaneous, the procedure is versatile and simple to run, rodents require little pre-training except for habituation to the test arena, and there is no food or water deprivation. This final feature means that the results should be insensitive to manipulations that affect motivation. It is also easy to vary task difficulty by altering the interval between sample and test. Furthermore, the task is well suited to mice as well as rats. The popularity of the spontaneous object recognition task is reflected in the fact that the initial paper by Ennaceur and Delacour [16] has been cited over a thousand times (ISI, Web of Science). It has also been estimated that approximately 43,000 animals have been used in this type of task or its close variants (see below) in the years 2007–2012 [25].

Ease of testing is not, however, sufficient justification for the adoption of a behavioural task. A far more important issue is whether the task has construct validity, i.e., that it tests the cognitive processes that are thought to support recognition memory and, as a consequence, relies on the same neural substrates. It could, for example, be argued that the spontaneous recognition test merely measures habituation to repeated stimuli, a form of implicit learning, and so is not comparable to the explicit tests of recognition memory given to humans. In fact, studies with rodents have shown that perirhinal lesions can leave intact the decrease in exploration that goes with repeated presentation of the same stimulus, i.e., habituation, but still impair object recognition based on the preference between two objects presented simultaneously [26–28].

A further set of issues concerns the number of processes that might contribute to recognition memory. One potential process is perceptual fluency, a form of implicit memory [29]. A related set of concerns arises from current models of human recognition memory, many of which assume the existence of two separate forms of information that can support recognition decisions [30,31]. These ‘two-process’ models typically presume the existence of ‘familiarity-based recognition’ and ‘recollective-based recognition’, which are often thought to have distinct neural substrates [31–33]. Recollection-based recognition is seen as the more complex as it involves additional information related to the target object. It is, therefore, argued on grounds of parsimony that animal tests of recognition memory should essentially tax familiarity-based recognition [34], i.e., they do not capture the full complexity of human recognition memory. As a consequence, novel, more elaborate, procedures are required to assess analogues of recollective-based recognition [35,36].

In spite of these issues, it is possible to determine if spontaneous tests of recognition memory tax similar neural structures to those required for forced-choice tests of recognition. One source of validation evidence comes from comparing the outcome of DNMS experiments with those using spontaneous object preference tasks. Taking the example of selective brain lesions in rats, it can be seen that perirhinal cortex lesions impair object recognition whether tested using spontaneous tasks [4,37–39] or DNMS procedures [40]. Likewise, perirhinal lesions in monkeys disrupt both DNMS and visual-paired comparison, the latter task compares the times spent looking at novel and familiar stimuli [41]. Similarly, lesions in sites such as the fornix, medial prefrontal cortex, and mammillary bodies spare object recognition in rats whether tested using nonmatching-to-sample [42,43] or spontaneous preference tests [17,20,39,44,45]. Comparisons between the consequences of hippocampal lesions in rats are more difficult to interpret as the majority of both spontaneous and reinforced nonmatching studies describe sparing of recognition memory, though other studies report deficits [4,46–53]. There is, however, evidence from studies of both monkeys and humans that hippocampal lesions can be more disruptive to visual paired comparison than DNMS tests of visual recognition memory [41,54,55]. Taken overall, spontaneous preference tests of recognition memory for rodents give comparable results to those found with reinforced nonmatching procedures; although for paired viewing studies with primates involving hippocampal lesions there may be added factors that explain the apparent discrepancy (see Section 4).

Since the introduction of the spontaneous object recognition test [16], researchers have devised ingenious variants, although the underlying logic of these tasks has remained largely unaltered. For example, rather than use a circular or square arena, some researchers have used a Y- maze, in which the two arms are used first for the sample phase and then for the recognition choice phase [51,56]. The rationale is that the apparatus ensures that the animals are placed in close proximity to the separate test stimuli, so increasing levels of exploration and making the assessment of relative exploration easier. The greater restriction on the rodent's location within the Y-maze has also made it possible to test recognition memory in the dark as well as cross-modal object recognition [57,58]. In another variant (Fig. 1E), rats are allowed to explore an E shaped maze in which objects are placed in the two outer arms where they cannot be seen directly from the start point [35]. This arm arrangement means that the rat makes its object choice without directly comparing objects, so creating additional recollective-like mnemonic demands [35,59].

#### 2.2.1 Spontaneous object recognition – shortcomings

Some of the very features that make the spontaneous object recognition task so attractive are the same features that bring potential problems. One intrinsic problem is that the task is based around spontaneous behaviour. This feature inevitably contributes to variance between subjects, so decreasing statistical power. A further issue is that the amount of exploration given to a particular novel or particular familiar object might be biased by individual preferences to specific types of objects. The solution is to counterbalance the choice of novel and familiar objects within a study, but this arrangement still adds variance, unless the test objects are equivalently matched for their attractiveness. A consequence of the inter-animal and inter-stimulus variance is the frequent need for additional trials, additional rodents, or both.

Another problem is that spontaneous recognition tasks use the differential exploration of novel and familiar objects to measure recognition. This form of measurement leads to issues of how best to define ‘exploration’, e.g., how close must be the animal, whether there are different intensities of exploration, and whether we should measure micro-behaviours associated with exploration. Studies adopt different criteria when measuring exploration, i.e., there is no agreed standard. The development of more automated analyses of exploration behaviour may prove important in this regard [60]. A further problem is the extent to which the test animal is stressed by being placed in an open arena and how that affects object exploration and discrimination [61,62]. For example, object recognition memory consolidation and retrieval in an open field have been shown to be modulated by post-training administration of the stress hormone, corticosterone, but only when the rats were not previously habituated to the arena [63]. Additionally, a stressful experience before the retention test can impair recognition memory at longer retention intervals (hours) but not at 5 min [64,65]. These issues gain added significance if stress or anxiety is affected by the neural manipulation under investigation. A potential confound also emerges if different groups vary in the time taken to accumulate a predetermined amount of sample object exploration [47]. Furthermore, as the task is based on differential exploration, the neural manipulation being tested should not have any sensorimotor effects, e.g., hyperactivity. This can be an important interpretational issue not only for systemic drug studies but also for certain lesions, e.g., for the hyperactivity that often accompanies hippocampal lesions [66].

Here, we will focus on two related problems associated with the standard spontaneous task. The first is that the length of time taken to run a single recognition test means that experimenters typically complete no more than one trial per day. This limitation means that test sessions often have to be repeated to combat variance. It also means that added importance is attached to the particular choice of the individual objects used for the familiar and novel stimuli. The second problem is that the test animals are repeatedly handled, not just before and after testing, but also in the middle of testing (to begin and end the retention interval). The individual reaction of the test animals to being held is again likely to increase variance, especially as both individual rodents and individual experimenters may behave differently. This problem is compounded further if the brain manipulation under investigation affects stress or affect. To counteract both problems it is often necessary to use relatively large group sizes or risk the problem of having insufficient power to detect real effects.

#### 2.2.2 Spontaneous object recognition – some solutions to these shortcomings

**Fig. 2:**
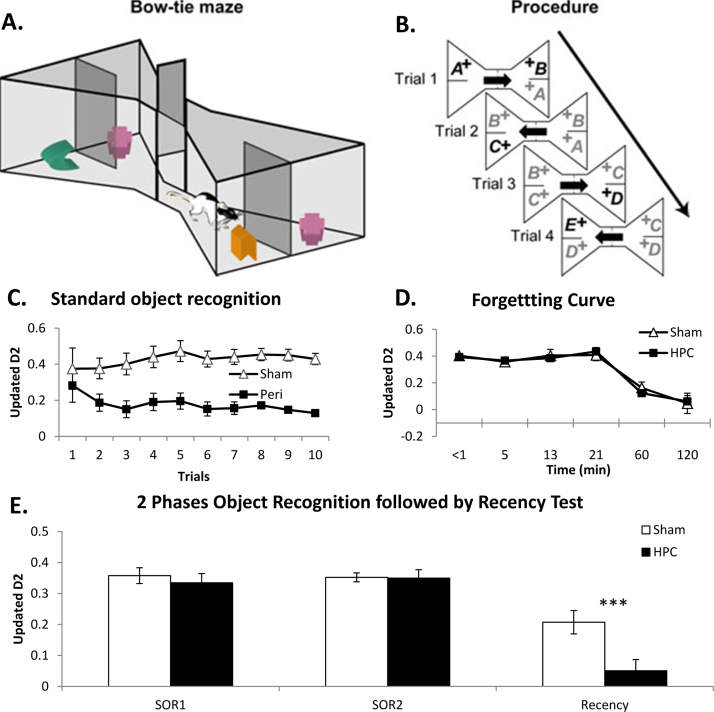
Bow-tie maze with associated behavioural data. (A) Schematic illustration of the bow-tie maze [67]. A central sliding door separates the two ends of the maze in which two objects are placed. (B) General procedure for the standard running object recognition test showing the presentation order of the objects. All objects are rewarded (+). Arrows show direction of rat movements. Bold letters represent novel objects and grey letters represent familiar objects. (A) and (B) adapted from [68]. (C) Object recognition by rats with perirhinal cortex lesions (black square) and surgical controls (white triangle) [27]; graph shows the updated D2 scores over successive trials. D2 is the time exploring the novel object minus the time exploring the familiar object, divided by total exploration. Scores can range from +1 to −1. (D) Object recognition forgetting curve: Graph shows updated D2 scores of composite object recognition memory performance of rats with hippocampal lesions (black square) and their controls (white triangle) across various retention intervals used in separate experiments. (E) Object recency: histogram showing the mean performance of rats with hippocampal lesions (black) and their surgical controls (white) on recency discrimination performance. Only the control group performed above chance. In addition, recognition performance is given for the two blocks of stimulus familiarisation trials (SOR1 and SOR2) that incorporate an object recognition test (retention delay 1 min). (D, E) Adapted from [69]. Data shown are mean ± standard error. Group differences ***p < 0.001.

There is a need to devise a task that utilises the strongest features of the spontaneous object recognition task while addressing as many of its shortcomings as possible. The ‘bow-tie maze’, introduced by Albasser et al. [67], was designed for this very reason. This task is a hybrid of DNMS and spontaneous object recognition, drawing key elements from both tasks. The central feature is that rodents repeatedly explore pairs of objects at opposite ends of an enclosed maze shaped like a bow-tie. Each pair of stimuli consists of one novel object and one familiar object (Fig. 2A and B). A sliding door in the middle of the maze separates the two ends of the maze, so ensuring discrete trials. This arrangement makes it possible to run multiple trials within a session without handling the rodents. Although the animals have to be pre-trained to run from one end of the maze to the other for food rewards, which adds to the labour involved, this pre-training helps to ensure that the animals are well habituated to the test environment and so reduces stress.

Food rewards are placed under (or nearby) the test objects to encourage their inspection and to ensure that the rats (or mice) travel up and down the apparatus. The animals do not, however, learn a reinforced nonmatching (or matching) rule as both novel and familiar objects are equally associated with reward [67]. Instead, recognition is still signalled by spontaneous exploration preferences. Each trial is typically just one minute long, i.e., much shorter than a normal spontaneous recognition trial. This time saving is possible because the rodents approach the objects almost immediately in order to retrieve food rewards, and so explore from the outset of the trial. The 1 min-trial time also takes advantage of the finding that in standard spontaneous object recognition tasks the most discriminatory period of exploration between novel and familiar objects often takes place at the beginning of the test session [18]. Exploiting these features, a rat in the bow-tie maze can, for example, receive six recognition trials in 7 min. In contrast, the same duration of testing in a standard spontaneous exploration task would normally allow just one trial. In practice, rats can readily receive 20 recognition trials in a single bow-tie maze session (in 21 min).

The bow-tie maze has high plain sides to limit distracting visual stimuli and reduce spatial cues (Fig. 2A). An overhead camera records the animal's movements, including exploration bouts. To begin a session (trial 0), the rat is put into one end of the maze, which also contains an object (A) that covers a food reward [67]. The animal is allowed to retrieve the food reward. One minute after being placed in the maze, the central sliding door is opened and the animal runs to the opposite end for more food rewards. In the simplest task design, the rodent finds novel object B and an identical copy of the now familiar, object A (trial 1). Recognition is reflected in the greater preference for novel object B. After a further minute, the central door is raised and the animal runs back to the opposite end, where it can explore object B (now familiar) and novel object C (trial 2). Recognition is measured by calculating the cumulative difference in time spent exploring the novel and familiar objects over successive trials (‘cumulative D1’). In addition, this D1 score can be divided by the total amount of object exploration to give the D2 index score [16]; a ratio that ranges between +1 and −1 (Fig. 2C).

Experiments run in the bow-tie maze have typically used this ‘running recognition’ protocol (Fig. 2B). The design not only increases the numbers of trials that can be given within a set period, as there is no discrete sample phase, but has another, more subtle, benefit. By ensuring that every object serves as both a familiar and a novel item, the influence of any individual object that might be particularly attractive or aversive to the test animals becomes nullified as any such effects should be counteracted across subsequent trials. A further benefit is that over the course of several sessions the task might use over a hundred different objects, rather than repeatedly using a very limited sample of objects that may have been selected because they give reliable recognition. Again, the increase in the number of stimuli helps to remove any biases associated with particular stimuli. Using these designs it has been found that perirhinal cortex lesions impair object recognition (Fig. 2C) while hippocampal lesions appear to spare recognition memory (Fig. 2D and E) [27,68–70]. This pattern is consistent with many studies using spontaneous object recognition (see Section 2.2).

The bow-tie maze offers many procedural variants to test different aspects of memory. Retention delays can be increased by delaying the repeat of a stimulus to nearer the end of the series of continuous trials (short retention delays, Table 1). In this way, both interference and retention delays can be increased by interposing other trials before returning to the now familiar object. Consequently, forgetting curves can be derived from the results of a single session [67] (Fig. 2D). An alternative approach is to divide the test session into two stages. In the first stage the rat is exposed to multiple stimuli, but then removed from the apparatus so that the test stage can follow at whatever interval is required (long retention delays, Table 1). The test stage then contains multiple trials at the pre-selected retention delay (see Table 1). Additionally, identical pairs of objects, either novel or familiar, could be presented before recognition testing in order to increase the amount of time (and interference) between discrimination trials or to further familiarise the animals with a particular set of objects. The latter procedure, involving repeat presentations, might be needed for overnight retention intervals.

The fact that the test objects are immediately adjacent to food rewards means that the rats are encouraged to approach the objects, which are in constant locations. The latter feature means that it is just as easy to test rodents in the dark as in the light, making it possible to examine nonvisual object recognition [27,70]. The bow-tie maze also makes it simple to test recency memory as well as recognition memory [69]. Indeed, using the protocol shown schematically in Table 1 it is possible to test both recognition and recency of the same objects within a single test session [69]. Although the preferred behavioural procedure within the bow-tie maze has been a running recognition design, discrete sample and test phases could readily be run at opposite ends of the apparatus, i.e., more akin to DNMS. The apparatus can also be used for object-in-place recognition [71]. Despite the ability to run these various tests of associative recognition, the apparatus has not, so far, been used for those tests that combine ‘where’, ‘what’ and ‘when’ information into the same trial [22–24], i.e., it may not lend itself to recollective-like tasks.

An immediate benefit of the bow-tie maze is its reliability in generating recognition discrimination scores (D1 and D2) that are significantly above chance with modest sized groups of animals [64]; see also [25]. The multiple trials mean that individual control rats almost invariably perform above chance with short retention delays [67]. A closely related feature is that the variance of the updated D2 scores for any group of animals decreases as the trial numbers within a session increase (Fig. 2C) [27,67]. Recent (unpublished) modifications have been made to the apparatus to reduce further any unintended distractions for the tests animals. For this reason, a ceiling has been added to the apparatus, along with doors at both ends of the maze through which objects and food rewards can be added or removed. In this way, spatial cues and extraneous noise can be limited, while the experimenter remains invisible from the subject between trials.

One possible shortcoming is that it may be more difficult, but not impossible [71], for rodents to combine space and object information within this apparatus. Another issue is that the standard test protocol for the bow-tie maze does not involve comparisons between pairs of novel stimuli and separate pairs of familiar stimuli [72]. This modified procedure, which has been used in a Y-maze, was introduced to determine if rats with perirhinal cortex lesions treat novel stimuli as though they are familiar or treat familiar stimuli as if they are novel [72]. In that study it was concluded that perirhinal cortex lesions cause false memories, i.e., that novel stimuli are treated as if familiar. In fact, the bow-tie maze very readily lends itself to comparable experiments in which rodents are presented with pairs of only novel or only familiar objects at the ends of the maze (see [68]). When this manipulation was examined in the bow-tie maze, no evidence could be found that rats with perirhinal lesions showed unusually low levels of exploration to pairs of novel objects [68].

The gain in power provided by hybrid tasks that combine features of DNMS and spontaneous object recognition, such as the bow-tie maze, has been examined more formally by Ameen-Ali et al. [25]. These researchers devised a slightly more elaborate task that shares many of the key features of the bow-tie maze. In their task there are several compartments but, like the bow-tie maze, the rat is trained to run through the apparatus where it receives separate sample and test trials without being handled. The animal receives multiple trials, e.g., 30 in a session [25]. The test objects are again associated with food rewards, while recognition is determined on the basis of preferred exploration. Comparisons with previous spontaneous object recognition studies by the same group helped to confirm the gain in statistical power associated with such hybrid methods [25].

A key advantage of these new task variants is the ability to give multiple test trials within a single session. The final sections describe investigations into the study of recognition memory that could not be conducted without this particular feature.

## 3 The functional imaging of rodent recognition memory: Immediate-early gene mapping

The term immediate-early gene (IEG) is reserved for the particular group of genes that do not require previous protein synthesis to be activated [73]. For this reason, they have a temporal advantage over other genes, so giving the term ‘immediate’. There are numerous immediate-early genes, which can be categorised into two groups. One group, the ‘regulatory transcription factors’, can influence cell function through the downstream genes that they regulate. The second group, ‘effector factors’ can directly control specific cellular functions. There are thought to be between 10 and 15 IEGs that are regulatory transcription factors [74]. Two of these are the genes c-*fos* and *zif268*, both of which are assumed to have roles in long-term plasticity [75–77].

The activity of the IEG c-*fos* is often used as an indirect marker for neuronal activity. This gene is widely distributed through the brain, and events that bring about increased neuronal activity will upregulate c-*fos* in numerous (but not all) brain sites [73,78]. The activity of this gene is particularly appropriate for studies related to specific aspects of behaviour as c-*fos* has low resting levels of activity that rapidly increase and then decrease back to baseline after an intervention [78,79]. This steep temporal profile reflects the autoregulation shown by this gene. The activity of c-*fos* has repeatedly been linked to plastic processes associated with learning and memory [76,77,80–83].

### 3.1 Comparing c-*fos* expression for novel and familiar stimuli

When rats see novel visual stimuli there is increased expression of c-*fos* in the perirhinal cortex [84–88], which parallels the increased neuronal activity found in the same area [89]. These findings are informative as the perirhinal cortex is known to be vital for normal visual recognition memory [4,32,40]. Of particular significance is the finding that blocking c-*fos* expression in the rat perirhinal cortex disrupts the stabilisation of long-term recognition memory [90]. For these reasons, expression of c-*fos* in the perirhinal cortex is seen as a marker for neural activity closely involved with recognition memory, creating the potential for new imaging approaches to understand this form of memory.

Although immediate-early gene imaging provides exceptional anatomical resolution (down to individual neurons) it has much poorer temporal resolution. For example, in experiments that examine levels of Fos protein, there is often a gap of around 90 min between the target learning behaviour and the sacrifice of the animal. While this interval is designed to capture peak production of Fos [76,79] it inevitably means that the source of the signal may become blurred. A further issue is that most IEG imaging studies require a control group that is matched for sensorimotor demands but is expected to show little or no learning when compared with the experimental group. Differential Fos levels are then assumed to reflect the learning condition. Clearly the validity of this subtraction method depends on the appropriateness of the control condition.

Initial studies of c-*fos* expression simply compared IEG activity levels in rats shown either novel stimuli or shown familiar stimuli [86–88]. These studies found raised c-*fos* activity associated with novel stimuli in the perirhinal cortex and visual association area Te2, but not in the hippocampus [86–88]. A refinement (the ‘split-viewing’ procedure) involved presenting novel visual stimuli to one eye of the rat and familiar stimuli to the other eye of the same rat [84,85,91]. Inter-hemispheric comparisons again showed that viewing novel stimuli was associated with raised c-*fos* expression in the perirhinal cortex and area Te2, but not in the hippocampus (Table 2) [84]. Changes in hippocampal activity were found, however, when stimulus novelty was introduced by rearranging the spatial configurations of familiar groups of stimuli. Now, hippocampal c-*fos* changes were found in the stimulus rearrangement group (Table 2) [84,92].

All of the c-*fos* studies cited so far suffer from the problem that there was no concomitant behavioural evidence to show that the rats could actually distinguish the novel from the familiar visual stimuli. It might, therefore, seem natural to repeat these experiments using spontaneous object recognition tasks. In fact, using the standard spontaneous recognition task [16] would be hugely problematic. In that task, animals normally experience a very small number of novel stimuli within a test session (often just one), making it unlikely that the neural signal would be sufficiently large to be detected. There may also be individual biasing effects caused by the particular objects selected for the task. Problems would also occur with individual animals that fail to show a clear preference for the novel stimuli. The need to handle the rat repeatedly would add further noise to the c-*fos* signal. The solution is to use a task that delivers multiple stimuli within a single recognition session, so increasing the signal strength, while also increasing the likelihood of deriving clear preference measures for novel over familiar stimuli for each individual animal.

The bow-tie maze provides a means to examine c-*fos* expression associated with recognition memory [70,93]. In studies using this apparatus, rats have been given 20 recognition trials, i.e., 20 novel objects vs 20 familiar objects, and then perfused 90 min later for the immunohistochemical visualisation of the Fos protein. Two aspects of this procedure merit particular consideration. The first is that the recognition test must contain both novel and familiar stimuli to make it possible to behaviourally confirm the recognition of repeated stimuli, i.e., the test cannot solely contain novel stimuli. The second is the issue of how best to design a control condition that isolates those changes in c-*fos* activity associated with recognition memory. This control condition needs to be matched to the visuo-motor demands of the recognition condition. In the first study to examine c-*fos* expression associated with behavioural measures of recognition [93], the control rats were given the same 20 recognition trials with the same set of 20 objects as those given to the experimental (recognition) group on the final test day. The critical difference was that these control rats had repeatedly been exposed to the same set of 20 objects over numerous, previous sessions, ensuring that all stimuli were familiar on the final test day. The impact of this familiarisation procedure could be seen in the final test session. The recognition memory group showed a strong preference for the novel over the familiar stimuli. In contrast, the familiar object control group showed no clear preference between the test objects, presumably reflecting their acquired familiarity [93].

Comparisons of c-*fos* expression after recognition testing in the bow-tie maze (novel object recognition condition versus familiar object control condition) revealed that recognition was associated with raised Fos counts (Table 2) in the caudal perirhinal cortex (areas 35 and 36), as well as area Te2 [93]. Other sites such as the prelimbic, infralimbic and anterior cingulate cortices did not show differential Fos levels. These results are very similar to those from c-*fos* studies in which rats were passively shown either novel or familiar stimuli [84–86], as well as paralleling the outcome of lesion studies in these same areas [4,45,94,95].

At the same time, there is one striking difference; hippocampal changes in c-*fos* expression were found in the bow-tie maze task, but had not been found in previous procedures that had merely presented novel stimuli (e.g., [84–86]). Comparisons between rats in the bow-tie maze exploring novel objects or only exploring familiar objects (Table 2), revealed that in the novel object group the hippocampal subfields CA3 (septal) and CA1 (temporal) show significant Fos increases, while the dentate gyrus (septal and intermediate) show a Fos decrease [93]. This pattern of hippocampal changes (Table 2) matches the Fos findings when rats are passively shown spatially rearranged familiar visual stimuli in the split-hemisphere procedure [84]. That is, relatively increased Fos counts were seen in CA1, while relatively decreased Fos counts were seen in the dentate gyrus [84,93]. One interpretation, to be considered in more detail later, is that by exploring objects in the bow-tie maze the rats not only showed differential neural responses associated with novelty versus familiarity, but also show additional neural changes arising from the learning of other information associated with individual objects, e.g., their spatial or temporal attributes [95].

In a complementary bow-tie maze study, c-*fos* expression was examined after rats had discriminated novel from familiar objects in the dark [70]. This study used essentially the same experimental and control protocols as described above (though all testing was in the dark). Thus, in the final session, one group experienced novel objects while the control group experienced the same set of familiar objects that had been given on all of the preceding sessions [93]. Comparisons between these two groups showed increased c-*fos* activity in rostral perirhinal cortex, but not in caudal perirhinal cortex, of those rats discriminating novel from familiar objects in the dark. This rostral-caudal perirhinal pattern is the opposite of that found for object recognition in the light [93], creating a potential double dissociation (Table 2). Novel objects in the dark were again associated with increased c-*fos* activity in the hippocampus, but the pattern of subfield change was also different to that seen the light (Table 2). In the dark there were significant Fos increases in the dentate gyrus, CA1 and CA3 [70], whilst in the light there was a Fos decrease in the dentate gyrus, as well as Fos increases in CA1 and CA3 [93]. In addition, a wider array of other brain regions, some involved in spatial memory, were activated by exploring novel objects in the dark in a bow-tie maze, e.g., the anterior thalamic nuclei, retrosplenial granular cortex, anterior cingulate cortex, and lateral entorhinal cortex.

A third c-*fos* imaging study examined neural activations associated with recency memory, i.e., the ability to distinguish temporal properties of stimuli [96]. The Recency Test group were allowed to discriminate between pairs of objects that had been previously explored either 110 min or 220 min earlier. The Recency Control group received pairs of different objects that had previously been presented one immediately after the other, ensuring that they could not be subsequently discriminated on this dimension. Given that both conditions involved recency memory, albeit at varying degrees of temporal resolution, it is not particularly surprising that there were no evident group differences in c-*fos* expression levels across the regions of interest [96]. There were, however, significant correlations in the Recency Test group between Fos levels and recency memory performance in multiple sites, including the perirhinal cortex, lateral entorhinal cortex, and several hippocampal subfields. These correlations were not found in the Recency Control condition.

### 3.2 Network analyses based on structural equation modelling (SEM)

A behavioural contrast that has been employed in several of these c-*fos* expression studies [70,93,96] is between rats that discriminate between novel and familiar objects (recognition memory) and rats that only explore familiar objects. The next question was, therefore, whether this qualitative difference is reflected by changes in the networks of activity across medial temporal lobe structures. To examine this prediction, an additional form of statistical analysis is required.

For these network analyses, the relationships between the Fos counts in different brain sites were examined using structural equation modelling (SEM). Structural equation models are multiple-equation regression models that can quantify causal (structural) relationships between a set of variables. These relationships include inferring the potential direction of influence between two regions. The strength of a relationship (path) between regions is estimated based on the regional Fos count covariance matrix. A model is assessed on how well it replicates the covariance matrices of the observed Fos data. The goal was to derive the best fitting models to explain the inter-correlations based on the covariance matrix for the patterns of activation seen between the various regions of interest [93]. The SEM models involved sites known to be important for recognition memory, most notably the perirhinal cortex, and were then applied to established anatomical pathways between regions of interest within and beyond the temporal lobe.

**Fig. 3:**
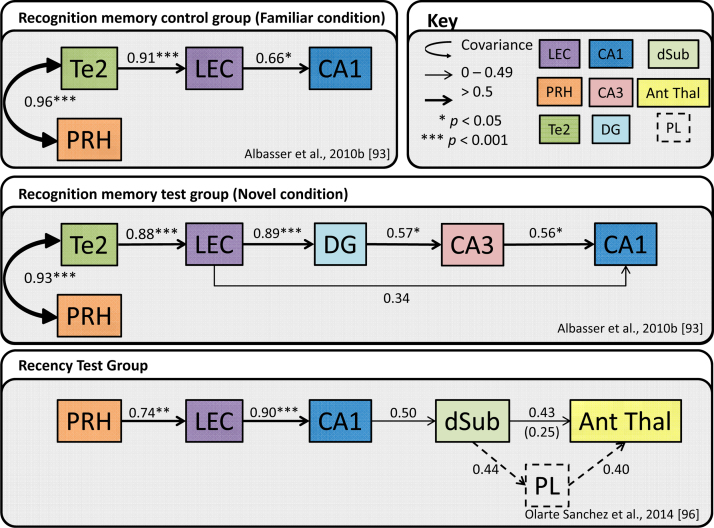
Neural networks derived for recognition and recency memory. Optimal interactions derived from structural equation modelling of Fos counts in the control familiar object condition (top panel) and novel object condition (middle panel); adapted from [93]. Optimal interactions derived for recency memory (lower panel); the dashed pathways involving the prelimbic cortex have been added to the model as these provide a further model with good fit. The number in brackets is the path coefficient when the prelimbic cortex is added to the model; adapted from [96]. The strength of the causal influence of each path is denoted both by the thickness of the arrow and by the path coefficient next to that path. Sites depicted: area Te2 (Te2), perirhinal cortex (PRH), lateral entorhinal cortex (LEC), hippocampal subfields CA1, CA3 and dentate gyrus (DG), dorsal subiculum (dSub), anterior thalamic nuclei (Ant Thal) and prelimbic cortex (PL). *p < 0.05; ***p < 0.001.

By applying SEM to the Fos counts from the initial bow-tie maze study of recognition memory, two different patterns of correlated activity emerged [93]. These patterns depended on whether the rats had explored novel or familiar objects. The optimum SEM model of correlated activity associated with exploring *novel* objects (Fig. 3, middle) involved area Te2, parahippocampal regions (perirhinal cortex and lateral entorhinal cortex), as well as various hippocampal subfields [93]. The best fitting SEM model associated with exploring *familiar* objects (Fig. 3, upper) again involved area Te2, the perirhinal and lateral entorhinal cortices, but there was a crucial difference in the hippocampus (Fig. 3). While novel stimuli were associated with preferential activity correlations in the direct pathway from the lateral entorhinal cortex to the dentate gyrus (and CA3), familiar stimuli were principally associated with correlated activity in the direct pathway from the lateral entorhinal cortex to CA1, i.e., the familiarity network largely bypassed the pathway to the dentate gyrus [93]. These differences are striking as they suggest a different mode of hippocampal interaction when learning about novel as opposed to familiar stimuli (Fig. 3).

These different activation models for novel and familiar stimuli were re-assessed in a study [97] that again measured c-*fos* expression in groups of rats tested on either the novel object or the familiar object protocols in the bow-tie maze as described above (see [93]). The inter-area correlations for Fos counts could then be added to those from Albasser et al. [93]. These combined data models led to further improvements in statistical fit, with qualitatively different network models for the novel stimuli condition and the familiar stimuli condition. As before, the key difference between these models was in the pathway from the entorhinal cortex (see Fig. 3).

The inference is that stimuli signalled as being familiar activate the hippocampus in a way that is qualitatively different from that seen for novel stimuli. The familiar stimulus model could be further examined by looking at a closely related form of memory, recency memory. This term describes the ability to distinguish stimuli based on their temporal properties, i.e., how long ago in the past they were last encountered. To test recency memory it is necessary to use familiar stimuli, and so it might be predicted that the c-*fos* activity network associated with recency memory will preferentially involve direct connections from the lateral entorhinal cortex to CA1. This prediction was tested as previously described (Recency Test, see Section 3.1) in the bow-tie maze [96]. In the Recency Test condition, counts of Fos-positive cells in a number of sites, including perirhinal cortex, lateral and medial entorhinal cortex, and hippocampal fields CA1 and CA3, were found to be significantly correlated with individual levels of recency discrimination by the rats. When the c-*fos* data were analysed using SEM methods, the model with the best fit comprised the linear pathway from the perirhinal cortex → lateral entorhinal cortex → CA1 → the subiculum (Fig. 3, lower). Crucially, the model was consistent with the familiarity conditions described above in that the preferred route from the lateral entorhinal cortex to the hippocampus was to CA1 and not to the dentate gyrus or CA3 [96]. The apparent importance of the CA1 field links with data showing how interactions between the hippocampus and prefrontal cortex support recency memory in rodents [45,98]. Along with the subiculum, the CA1 field is the source of the direct hippocampal inputs to prefrontal cortex, a pattern of connectivity reflected in SEM models of recency memory that additionally include prelimbic cortex (see Fig. 3).

Other relevant evidence comes from a study that compared activity levels of a different immediate early gene, *zif268*, in the hippocampal and parahippocampal regions of rats. The learning task, spatial working memory in a radial arm maze, was selected as it is known to depend on the hippocampus [99]. In contrast to object recognition, task performance does not normally require the integrity of the perirhinal cortex [100]. In this experiment, *zif 268* levels associated with either early or late learning of a radial-arm maze task [101] were compared. The optimal SEM model associated with early learning (i.e., when there should be more novel information and larger gains of learning) was remarkably similar to that found for novel object recognition in the bow-tie maze (Fig. 3, middle). Consequently, early radial-maze learning was associated with entorhinal → dentate/CA3 interactions [101]. In contrast, late learning was more associated with direct entorhinal → CA1 interactions, while the dentate gyrus and CA fields now seemed functionally disconnected (Fig. 3, upper).

## 4 Implications of network models of medial temporal lobe IEG activity

The notion that a novelty signal from the perirhinal cortex affects hippocampal processing is predicted by the gatekeeper model of declarative function [102]. In that model it is assumed that a novel item will increase rhinal processing, leading both to a feeling that the item is unknown and enhanced transfer to the hippocampus for further encoding. Conversely, the more familiar is an item, the less perirhinal processing it requires and the less vigorously it will be encoded in memory [102]. The present SEM findings both concur and extend these notions by identifying potential anatomical substrates reflecting changes in hippocampal activity, depending on the novelty or familiarity of the stimulus being processed.

The behavioural significance of such mechanisms is indicated by reference to learning theory. An influential assumption is that novel stimuli, which have uncertain consequences, attract more attention and enhanced rates of learning about their associated properties than familiar stimuli [103,104]. As already described, signals in the perirhinal cortex can provide information about the novelty or familiarity of a discrete stimulus, such as an object. Furthermore, lesion studies show that the perirhinal cortex operates in concert with the hippocampus to ensure that associated information, such as its location, is automatically acquired for a given object, i.e., ‘object-in-place’ information [45,48,95]. The immediate-early gene expression studies described in Section 3 reveal that novel stimuli are associated with the engagement of pathways from the entorhinal cortex to the dentate gyrus and CA3 (‘perforant pathway’) and, thence, to CA1 [93,96,97,101]. These hippocampal pathways would then be engaged to aid the rapid learning of new object-in-place information. This model fits with an array of other evidence implicating the dentate gyrus in pattern separation and the CA3 field in pattern completion [105–108]. Pattern separation, in particular, seems particularly relevant for distinguishing stimuli with novel features. The significance of this process can be seen in the assumption that the learning of associated information is more rapid for novel than familiar stimuli [104]. Pattern completion, meanwhile, provides a check against what might be already known, so both avoiding redundancy and helping to access prior learning experiences.

In contrast, the SEM analyses indicate that familiar stimuli are associated with the direct pathway (‘temporoammonic’) from the entorhinal cortex to CA1, a hippocampal subfield closely linked with temporal discriminations such as recency [109–112]. This function is especially pertinent for familiar stimuli. This distinction between the treatment of novel and familiar stimuli poses the question of why familiar stimuli do not appear to engage the CA3 field preferentially given this subarea's putative role in pattern completion, a function seemingly tailored to familiar components of incoming information. As noted above, a part of the explanation may reflect the need to reduce false positives for novelty. While this issue remains in need of further examination, relevant information comes from a recent study into the c-*fos* activation patterns in different lamina of the lateral entorhinal cortex [97].

The differential patterns of hippocampal activity for novel versus familiar objects imply a switch that originates from the parahippocampal region. It is, therefore, of much potential significance that entorhinal cortex layer III projects to CA1 while layer II projects to the dentate gyrus and CA3 [113,114]. This laminar segregation prompted an investigation of Fos levels within different entorhinal layers in order to determine whether the changes in hippocampal c-*fos* activity are reflected within the various entorhinal lamina [97]. The resulting well-fitting SEM models for familiar objects involved layer III (but not layer II) of the lateral entorhinal cortex, once again signalling the significance of the direct CA1 connections. A further issue concerns other pathways that might also be involved in a novelty/familiarity switching mechanism, most notably the direct hippocampal projections from the perirhinal cortex, which preferentially target the CA1 field and subiculum, potentially providing information on novelty/familiarity, as well as recency [95]. These perirhinal direct inputs are, however, thought to be appreciably lighter than the inputs via the lateral entorhinal cortex. In addition, hippocampal feedback upon parahippocampal regions could further bias network activity [115]. Such dynamic interactions between the perirhinal cortex and the hippocampus have been highlighted in human fMRI studies, which point to partial divisions of function across these same structures that relate to the recognition and recollection of associated information [116,117].

It is clear that our understanding of the neural basis of recognition memory has been enormously aided by the introduction of spontaneous preference tests for rodents. It must also be remembered that the results from these tasks can sometimes prove difficult to interpret as there are potential confounds. A particular, longstanding problem concerns the inconsistent effects of hippocampal lesions in rodents on object recognition [4,47,52]. While deficits are found in some spontaneous preference experiments, many others find no impairment, leading to a confusing literature. Our recent SEM analyses may provide a potential explanation for this inconsistency. The first point is that for some spontaneous tests of object recognition, associated item information that depends on the hippocampus (context, location in time or place) may contribute to the normal pattern of object discrimination. Consequently, hippocampal lesions may sometimes alter levels of preference without disrupting the underlying ability to initially detect novel or familiar stimuli. The second point arises from the fact that spontaneous recognition tests are typically based on comparisons between the total times spent exploring objects and not, for example, the first choice of object to be selected by the rodent. In normal animals, exploration of novel objects may prove to be prolonged by the engagement of hippocampal subfields that help to determine whether any prior learning about the stimulus has occurred and to ensure the effective learning of new associated information [55]. The loss of this initial exploration could be interpreted as a recognition memory deficit, despite the animal being able to discriminate novel from familiar stimuli.

In summary, spontaneous tests of object recognition have proved remarkably powerful, despite their inherent limitations and potential confounds. These problems include the need to give careful consideration to whether the initial sample exploration phase (Fig. 1C) is affected by the manipulation under investigation. More attention should also be given to the detailed nature of any exploratory bouts, rather than simply deriving an overall total of time spent exploring objects according to an arbitrary criterion [56]. Finally, there still remains a need to develop an effective DNMS-like task for rodents to complement the numerous studies using spontaneous exploration to assay recognition memory.

## Figures and Tables

**Table 1:** Sequences of object presentation used for three different types of object recognition memory study and an object recency study.

*Standard running recognition protocol*													
Trial	0	1	2	3	4	5	6	7	8	9	10	11	12
	–	A	B	C	D	E	F	G	H	I	J	K	L
	**A**	**B**	**C**	**D**	**E**	**F**	**G**	**H**	**I**	**J**	**K**	**L**	**M**

*Object recognition protocol with short variable delay*													
Trial	0	1	2	3	4	5	6	7	8	9	10	11	12
	–	A	B	C	D	E	F	E	E	D	C	B	A
	**A**	**B**	**C**	**D**	**E**	**F**	**G**	**H**	**I**	**J**	**K**	**L**	**M**

*Object recognition protocol with long variable delay*													
Trial	0	1	2	3	4	5		6	7	8	9	10	
	–	A	B	C	D	E	Variable	A	B	C	D	E	
	**A**	**B**	**C**	**D**	**E**	**F**	Delay	**G**	**H**	**I**	**J**	**K**	

*Recency protocol*													
Trial	0	1	2	3		4	5	6	7		8	9	10
	–	A	B	C	Delay	–	E	F	G	Delay	E	F	G
	**A**	**B**	**C**	**D**		**E**	**F**	**G**	**H**		**A**	**B**	**C**

Every trial consists of one novel object and one familiar object, each depicted by a letter, with the exception of Trial 0, which allows the initial object to become familiar. Novel objects are indicated in bold type. The length of time between initial exposure to an object and its subsequent use as a “familiar” object varied with the conditions. For the standard running recognition, each trial is 1 min. The delay to discrimination can be increased to several minutes using the short variable delay protocol or a long delay can be interposed between trials by removing the animal from the apparatus (long variable delay protocol). The recency protocol consists of two blocks of sample stimuli; the animal is removed from the apparatus after these blocks followed by a test phase in which an object from the first block is always paired with an object from the second block. Normal rats prefer to explore the item from further back in time.

**Table 2:** Summary table of c-*fos* expression showing the patterns of Fos changes in various types of recognition memory problem.

Brain region	Novel object bow-tie maze (light) [93]	Novel object bow-tie maze (dark) [70]	Paired viewing: Novel/familiar single images [84]	Paired viewing: Novel/familiar arrangement [84]
CA1	↑	↑	No change	↑
CA3	↑	↑	No change	No change
Dentate gyrus	↓	↑	No change	↓
Subiculum	No change	–	No change	↓
Lateral Entorhinal	No change	↑	No change	No change
Medial Entorhinal	No change	No change	–	–
Rostral Perirhinal	No change	↑	–	–
Caudal Perirhinal	↑	No change	↑	No change
Area Te	↑	No change	↑	No change

Symbols: ↑ signals increased Fos counts with novelty; ↓ signals decreased Fos counts with novelty. A horizontal bar (–) indicates that no Fos counts were made in that structure. Square brackets refer to references.
